# OPN‐Mediated Crosstalk Between Hepatocyte E4BP4 and Hepatic Stellate Cells Promotes MASH‐Associated Liver Fibrosis

**DOI:** 10.1002/advs.202405678

**Published:** 2024-10-29

**Authors:** Sujuan Wang, Jiashi Gao, Meichan Yang, Gary Zhang, Lei Yin, Xin Tong

**Affiliations:** ^1^ Department of Infectious Diseases The Second Xiangya Hospital Central South University 139 Renmin Middle Rd, Furong District Changsha Hunan 410011 P. R. China; ^2^ Department of Molecular & Integrative Physiology University of Michigan Medical School NCRC 20–3843, 2800 Plymouth Road Ann Arbor MI 48105 USA; ^3^ Caswell Diabetes Institute University of Michigan Medical School NCRC 20–3843, 2800 Plymouth Road Ann Arbor MI 48105 USA; ^4^ Department of Radiology Guangdong Provincial People's Hospital (Guangdong Academy of Medical Sciences) Southern Medical University 106 Zhongshan 2nd Road Guangzhou Guangdong 51008 P. R. China; ^5^ Provincial Key Laboratory of Artificial Intelligence in Medical Image Analysis and Application Guangzhou Guangdong 51008 P. R. China

**Keywords:** E4BP4, hepatic stellate cells, liver fibrosis, MASH, OPN

## Abstract

Stressed hepatocytes promote liver fibrosis through communications with hepatic stellate cells (HSCs) during chronic liver injury. However, intra‐hepatocyte players that facilitate such cell‐to‐cell communications are largely undefined. It is previously reported that hepatocyte E4BP4 is potently induced by ER stress and hepatocyte deletion of *E4bp4* protects mice from high‐fat diet‐induced liver steatosis. Here how hepatocyte *E4bp4* deficiency impacts the activation of HSCs and the progression toward MASH‐associated liver fibrosis is examined. Hepatic E4BP4 is increased in mouse models of NASH diet‐ or CCl4‐induced liver fibrosis. Hepatocyte‐specific *E4bp4* deletion protected mice against NASH diet‐induced liver injury, inflammation, and fibrosis without impacting liver steatosis. Hepatocyte E4BP4 overexpression activated HSCs in a medium transfer experiment, whereas hepatocyte *E4bp4* depletion did the opposite. RNA‐Seq analysis identified the pro‐fibrogenic factor OPN as a critical target of E4BP4 within hepatocytes. Antibody neutralization or shRNA depletion of *Opn* abrogated hepatocyte E4BP4‐induced HSC activation. E4BP4 interacted with and stabilized YAP, an established activator of OPN. Loss of hepatic *Yap* blocked OPN induction in the liver of Ad‐E4bp4‐injected mice. Hepatocyte E4BP4 induces OPN via YAP to activate HSCs and promote liver fibrosis during diet‐induced MASH. Inhibition of the hepatocyte E4BP4‐OPN pathway could offer a novel therapeutic avenue for treating MASLD/MASH.

## Graphical abstract

Stressed hepatocytes promote liver fibrosis by activating hepatic stellate cells (HSCs) during MASH. The study aims to determine the role of hepatocyte E4BP4 in communication with HSCs in liver fibrosis models. Hepatocyte E4BP4 induces OPN via YAP to activate HSCs and promote liver fibrosis. Inhibition of the hepatocyte E4BP4‐OPN pathway could offer a novel therapeutic avenue for treating MASH.

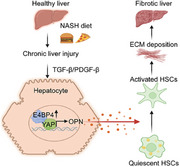

## 1 Introduction

Metabolic dysfunction‐associated steatotic liver disease (MASLD) affects ≈25–30% of the U.S. adult population. The progression of MASLD toward metabolic dysfunction‐associated steatohepatitis (MASH) is expected to become the leading cause of liver transplantation in the U.S.^[^ ^1^, ^2^, ^3^ ^]^ Fibrosis, a central feature of MASH, is excessive accumulation of extracellular matrix (ECM) due to overproduction and insufficient degradation of ECM.^[^ ^4^, ^5^, ^6^ ^]^ Hepatic stellate cells (HSCs) constitute the main source of ECM‐producing fibroblasts in models of diet‐induced MASLD/MASH.^[^ ^6^, ^7^, ^8^, ^9^ ^]^ In healthy liver, HSCs are quiescent and function primarily as the depot of retinoids.^[^ ^10^ ^]^ In response to liver injury or inflammation, they become activated with elevated expression of collagen, the main source of ECM accumulation.^[^ ^7^, ^9^ ^]^ The fibrotic process could be reversed once injury or inflammation is resolved.^[^ ^6^ ^]^ However, during chronic nutritional and inflammatory stress, HSC activation becomes persistent and amplified.^[^ ^7^, ^9^ ^]^ How to reverse HSCs activation becomes the main target for treating liver fibrosis.

Abundant evidence suggests cellular networks or cell‐cell communications in the liver regulate HSC activation and fibrosis development in MASH.^[^ ^11^, ^12^, ^13^ ^]^ So far, the cellular network involving hepatocytes, macrophages, and HSCs is the most important driver of fibrosis in MASH.^[^ ^12^, ^14^ ^]^ In particular, it has been shown that stressed and injured hepatocytes can trigger HSC activation by promoting inflammation, resulting in the recruitment of macrophages and their secretion of profibrogenic mediators such as TGF‐β and PGDF‐β.^[^ ^15^, ^16^, ^17^ ^]^ Meanwhile, there is also evidence in support of HSC activation through direct crosstalk between stressed hepatocytes and HSCs.^[^ ^12^, ^18^, ^19^ ^]^ It has been reported that hepatocyte‐derived signals can either directly activate the fibrogenic program of HSCs or serve as amplifying signals of the TGF‐β pathway.^[^ ^20^ ^]^ For example, hepatocytes were found to synthesize and release a number of profibrogenic factors including hedgehog ligands, Osteoponitin (OPN), BMP8B, and IL‐11 under stress conditions, and eventually promote HSCs activation.^[^ ^18^, ^19^, ^20^, ^21^ ^]^ However, whether targeting these hepatocyte‐derived profibrogenic factors could effectively reverse the progression of fibrosis remains to be tested.

First identified in bone tissue, OPN is a highly secreted glycol protein with high expression in liver tissue.^[^ ^22^, ^23^ ^]^ Work from Nieto's group provides concrete evidence of the role of OPN in liver regeneration and chronic liver diseases.^[^ ^22^ ^]^ OPN is essential for liver regeneration following partial hepatectomy, given that delayed regeneration occurs in *Opn* global knockout conditions.^[^ ^24^ ^]^ Serum and hepatic OPN levels are increased in patients with alcoholic fatty liver disease and MASLD with fibrosis.^[^ ^25^, ^26^ ^]^ *Opn^−/−^* mice fed methionine‐ and choline‐deficient (MCD) diet or high‐fat diet (HFD) with 2% cholesterol are protected from fibrosis but not from liver steatosis.^[^ ^25^, ^27^, ^28^ ^]^ In HSCs, OPN amplifies not only the proliferation of HSCs but also the TGF‐β‐mediated HSC activation.^[^ ^29^ ^]^ OPN was also found to increase collagen‐I synthesis via integrin‐CD44 and AKT signaling.^[^ ^24^, ^26^ ^]^ Since various cell types can produce OPN, it is intriguing to understand the role of hepatocyte‐derived OPN in modulating HSCs and fibrosis during diet‐induced MASH.

The b‐ZIP transcription factor E4‐binding protein 4 (E4BP4) is known for its role in NK cell development and immunomodulation.^[^ ^30^ ^]^ Our lab previously reported that hepatocyte E4BP4 is a critical regulator of lipid metabolism.^[^ ^31^ ^]^ Hepatic E4BP4 is induced by refeeding, insulin, and endoplasmic reticulum stress signals.^[^ ^32^, ^33^, ^34^ ^]^ Insulin‐induced E4BP4 in the liver is required to support SREBP‐1c‐mediated de novo lipogenesis.^[^ ^32^ ^]^ Moreover, hepatocyte E4bp4 deficiency protects mice from liver steatosis either after high‐fat‐low‐methionine‐choline‐deficient (HFLMCD) diet or HFD by impacting lipid metabolism and biosynthesis of lipid droplets.^[^ ^34^, ^35^ ^]^ Whether hepatocyte E4BP4 also impacts liver fibrosis during the progression toward MASH has not been examined.

In this study, we present evidence that hepatocyte E4BP4 promotes HSC activation and liver fibrosis via OPN‐mediated cell‐cell crosstalk. Hepatocyte *E4bp4*‐knockout mice are protected from NASH diet‐induced liver fibrosis and injury despite similar liver steatosis. In vitro medium transfer experiments demonstrate that overexpression of E4BP4 in hepatocytes activates HSCs in a hepatocyte OPN‐dependent manner. We also reveal that hepatic E4BP4 induces the OPN expression in part through its regulation of the YAP stability.

## 2 Result

### 2.1 Induction of E4BP4 in the Mouse Liver with Liver Fibrosis and in TGF‐β or PDGF‐β Treated Hepatocytes

**Figure 1:**
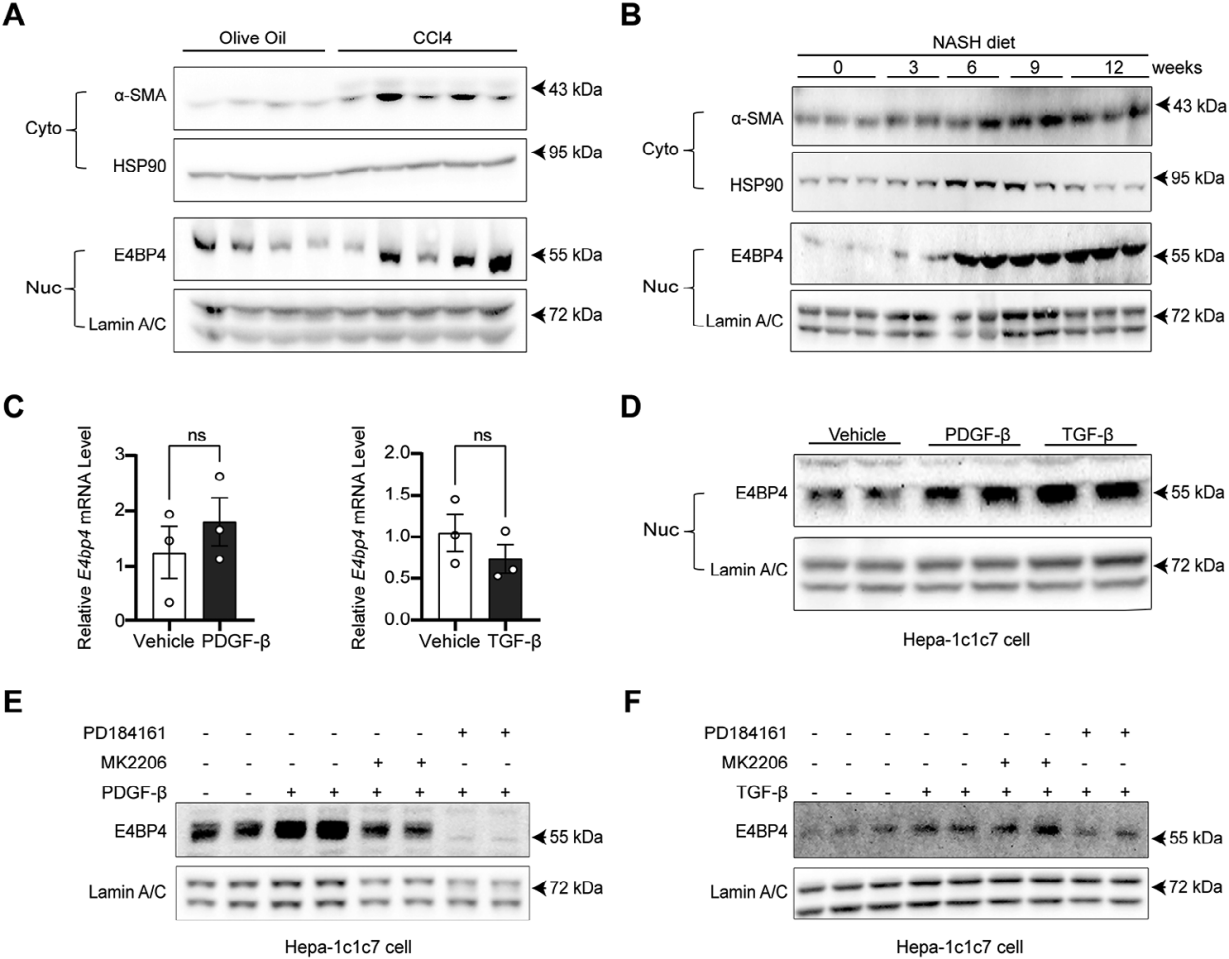
Induction of liver E4BP4 in either CCl4 or NASH diet‐induced mouse models of liver fibrosis. A) 8‐week‐old C57BL/6 WT male mice were subjected to intraperitoneal injection of either olive oil (*n* = 4) or CCl4 (0.6 µL g^−1^, 1:9 diluted in olive oil) (*n* = 5) for 8 weeks (twice a week) to generate chronic fibrosis model. Protein levels of the indicated proteins in liver lysates were measured by immunoblotting. B) 8‐week‐old C57BL/6 WT male mice were subjected to NASH diet for 0, 3, 6, 9, and 12 weeks prior to dissection at the same age. Protein levels of the indicated protein in liver lysates were determined by immunoblotting. C,D) Hepa‐1c1c7 were treated with TGF‐β (2 ng mL^−1^) or PDGF‐β (20 ng mL^−1^) for 16 h prior to examining mRNA levels of *E4bp4* by RT‐qPCR and the abundance of nuclear E4BP4 by immunoblotting. E) Hepa‐1c1c7 were treated with either vehicle, ERK inhibitor (PD184161, 30 nm) or AKT inhibitor (MK2206, 20 nm) 1 h prior to PDGF‐β (20 ng mL^−1^) treatment for 16 h. The abundance of nuclear E4BP4 was detected by immunoblotting. **F**) Hepa‐1c1c7 were treated with either vehicle, ERK inhibitor (PD184161, 30 nm) or AKT inhibitor (MK2206, 20 nM) 1 h prior to TGF‐β (2 ng mL^−1^) treatment for 16 h. The abundance of nuclear E4BP4 was detected by immunoblotting. The data were plotted as Mean ± SEM. ^*^ *p* < 0.05, ^**^ *p* < 0.01, ^****^ *p* < 0.0001 by the Student's *t*‐test.

We set out to explore liver E4BP4 expression in mouse models of chemical‐ and diet‐induced liver fibrosis. We used the induction of α‐SMA in liver tissue as a marker of liver fibrosis.^[^ ^7^ ^]^ As a potent liver fibrosis‐inducing hepatotoxin, carbon tetrachloride (CCl4) has been used to generate chemical‐induced liver fibrosis in mice.^[^ ^36^ ^]^ Following the 8‐week treatment of CCl4, hepatic E4BP4 protein levels were also increased, in parallel with the markedly elevated α‐SMA, a classical marker of liver fibrosis (**Figure** **1**A).^[^ ^37^, ^38^ ^]^ Next, we fed WT mice with NASH diet rich in saturated fat, cholesterol, and fructose to induce diet‐induced MASH^[^ ^21^ ^]^ and examined liver E4BP4 expression during feeding. In comparison with regular chow‐fed WT mice, NASH diet feeding elevated α‐SMA levels as early as 3 weeks, which continued to rise after 9 and 12 weeks. In the same set of liver samples, the E4BP4 protein was barely detectable in regular chow mice, whereas its level increased as early as 3 weeks of NASH diet feeding and stayed elevated throughout the entire feeding (Figure 1B). In summary, liver E4BP4 was shown to be induced in mouse models of liver fibrosis.

Since the liver consists of both parenchymal cells and non‐parenchymal cells, it is unclear whether hepatocyte E4BP4 is upregulated in response to fibrogenic stimuli. Several profibrogenic factors (TGF‐β, PDGF‐β, IL‐11, and IL‐17) play crucial roles in promoting HSC activation and ECM production.^[^ ^9^ ^]^ However, whether these profibrogenic factors could directly impact hepatocyte E4BP4 expression is unknown. Thus, we treated hepa‐1c1c7 cells with PDGF‐β or TGF‐β and examined the mRNA and protein levels of E4BP4. To our surprise, neither PDGF‐β nor TGF‐β affected the *E4bp4* mRNA levels (Figure 1C). In contrast, both PGDF‐β and TGF‐β elevated the nuclear E4BP4 protein abundance (Figure 1D), indicating a role of post‐translational modifications in enhancing hepatocyte E4BP4 after profibrogenic stimuli.

To gain new insights into the intracellular signaling pathways downstream of TGF‐β^[^ ^39^, ^40^ ^]^ and PDGF‐β^[^ ^41^ ^]^ on induction of E4BP4 protein, we employed the specific AKT inhibitor MK2206^[^ ^42^ ^]^ and ERK inhibitor PD184161^[^ ^43^, ^44^ ^]^ prior to TGF‐β or PDGF‐β treatment. Blocking ERK signaling but not AKT pathway abrogated TGF‐β‐mediated induction of E4BP4 protein (Figure 1E). Blocking either AKT or ERK signaling pathways impaired PDGF‐β‐mediated induction of E4BP4 (Figure 1F). Altogether, our data show the elevation of liver E4BP4, especially hepatocyte E4BP4, in various mouse models of liver fibrosis in response to profibrogenic factors including TGF‐β and PDGF‐β through the ERK signaling pathway.

### 2.2 Hepatocyte *E4bp4* Deficiency Protects Mice from NASH Diet‐Induced Liver Fibrosis

**Figure 2:**
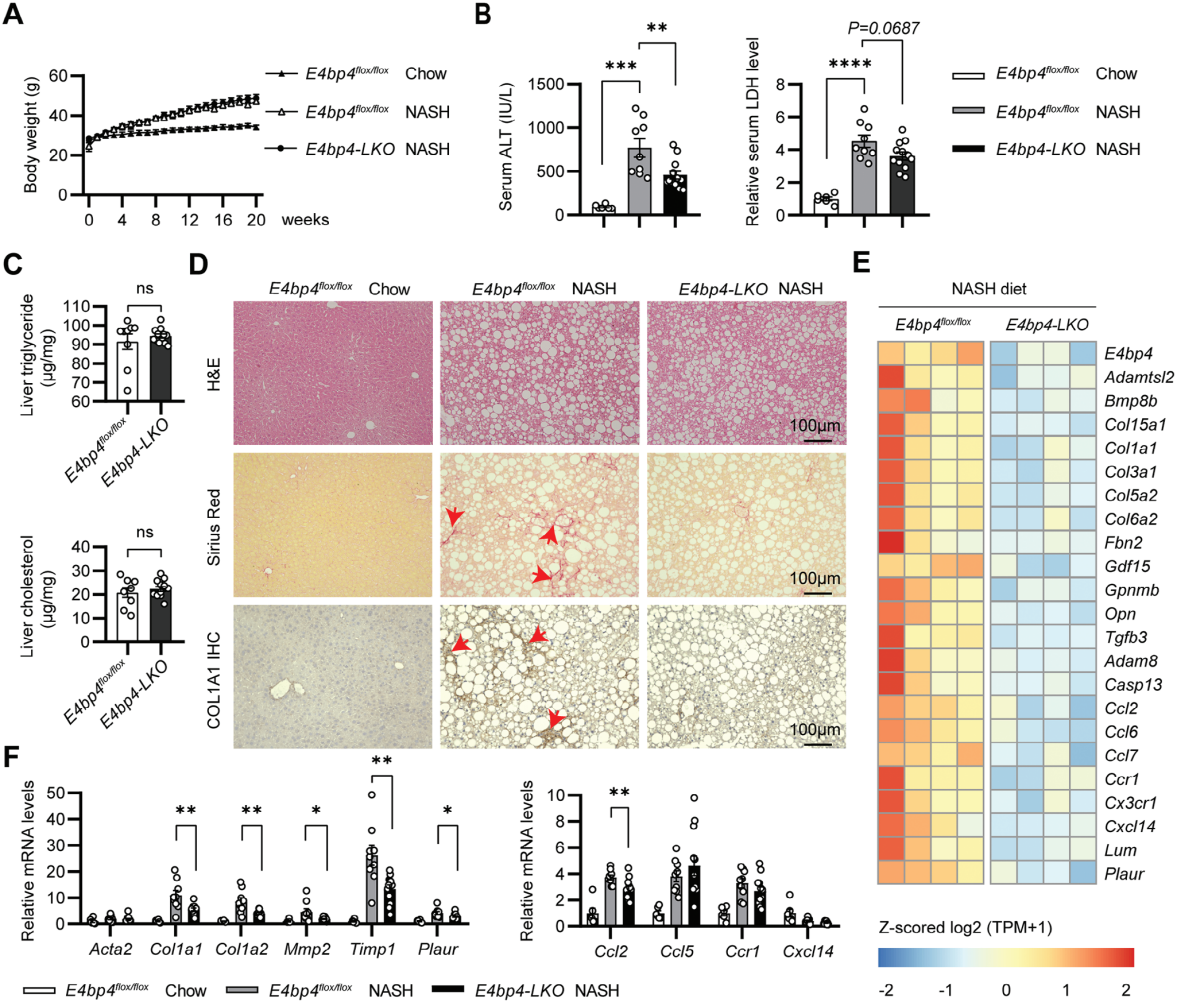
Hepatocyte *E4bp4* deficiency protects mice from NASH diet‐induced liver fibrosis independently of liver steatosis. Both 8‐week‐old *E4bp4^flox/flox^* male littermates (*n* = 9) and *E4bp4^flox/flox^* Alb‐Cre(+) (*E4bp4‐LKO*) male mice (*n* = 12) were fed with NASH diet for 20 weeks. *E4bp4^flox/flox^* male on the chow diet were used as control group (*n* = 6). A) Weekly body weight; B) serum ALT and LDH levels; C) liver triglycerides and cholesterol in NASH diet‐fed *E4bp4^flox/flox^* and *E4bp4‐LKO* mice; D) H&E staining, Sirius Red staining and Immunohistochemistry (IHC) with anti‐COL1A1; E) RNA‐seq analysis was performed using RNA samples from livers of NASH diet‐fed *E4bp4^flox/flox^* (*n* = 4) versus *E4bp4‐LKO* (*n* = 4) mice. The heatmap of the genes related to fibrosis and inflammation was shown; F) Liver samples of the three groups of mice were also used to assess the expression levels of fibrosis genes and inflammatory genes by RT‐qPCR.The data were plotted as Mean ± SEM. ^*^ *p* < 0.05, ^**^ *p* < 0.01, ^***^ *p* < 0.001 by one‐way ANOVA for (B) and (F); by the Student's *t*‐test for C.

To examine the potential role of hepatocyte E4BP4 in the development of liver fibrosis, we first fed liver‐specific *E4bp4* knockout (*E4bp4‐LKO*) mice and their littermate control mice MASH diet for 20 weeks. Both groups of mice had similar body weight gain by the end of NASH diet feeding (**Figure** **2**A). However, *E4bp4‐LKO* mice showed a significant reduction in serum ALT as well as a downward trend in another liver injury marker, serum LDH level,^[^ ^45^ ^]^ after NASH diet feeding (Figure 2B).

Unexpectedly, the liver TG and cholesterol levels were quite comparable between the two groups of mice (Figure 2C). Liver histology showed lipid accumulation in the liver of both groups of mice with the size of lipid droplets being relatively smaller in the liver of *E4bp4‐LKO* mice. More importantly, Sirius Red staining and COL1A1 immunohistochemical (IHC) staining revealed less ECM deposition in the liver of *E4bp4‐LKO* mice (Figure 2D). This observation was supported by RNA‐seq analysis with downregulated markers of liver fibrosis (*Col1a1, Gpnmb, Lum*) as well as inflammation (*Ccl2, Ccl6*, and Ccl7) (Figure 2E) in the liver of *E4bp4‐LKO* mice. We confirmed the downregulation of *Col1a1, Col1a2, Mmp2, and Timp1* by RT‐qPCR (Figure 2F). In contrast, we did not observe significant differences in lipid metabolic genes, including DNL, FAO, and lipid droplet binding proteins (Figure S1, Supporting Information). To address whether this is a sex‐specific phenotype, we compared female *E4bp4‐LKO* mice and their WT littermates on a 20‐week NASH diet. Unlike male mice, female *E4bp4‐LKO* mice exhibit a similar degree of liver injury by serum ALT and LDH as well as comparable liver steatosis by liver TG and cholesterol (Figure S2A,B, Supporting Information). At the molecular levels, the mRNA expression of fibrosis was similar between WT versus *E4bp4‐LKO* female mice (Figure S2C, Supporting Information).

Taken together, hepatocyte *E4bp4* deficiency in male mice blocks the progression of diet‐induced fatty liver toward MASH without impacting lipid metabolism and the development of liver steatosis. These findings also suggest that hepatocyte E4BP4 in female mice is dispensable during diet‐induced MASLD/MASH.

We also examined how the loss of hepatocyte E4BP4 impacts CCl4‐induced liver fibrosis since liver E4BP4 was potently elevated in WT mice injected with CCl4 (Figure 1A). Compared to WT mice, *E4bp4‐LKO* mice developed less liver injury and reduced Sirius Red and COL1A1 staining (Figure S3A,B, Supporting Information), consistent with the phenotype observed in the NASH diet experiment.

### 2.3 Hepatocyte E4BP4 Promotes Activation of Hepatic Stellate Cells via Cell–Cell Communications

**Figure 3:**
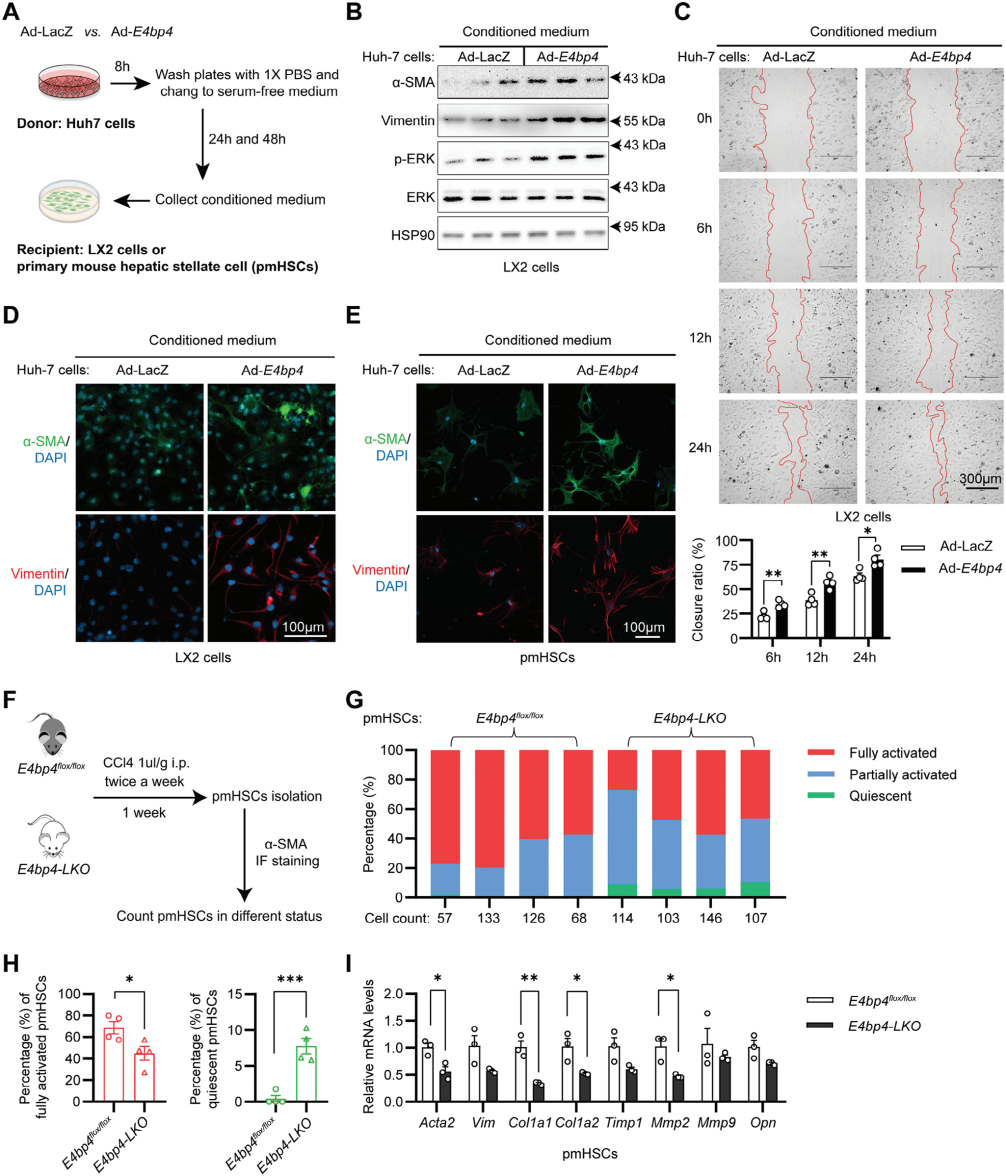
Hepatocyte E4BP4 promotes activation of hepatic stellate cells in vitro and in vivo. A) Schematic illustration of experimental design. B) LX2 cells were collected for immunoblotting to assess fibrosis markers 48 h after conditioned medium treatment. C) A wound healing assay was conducted to assess the ability of stellate cell migration 24 h postconditioned medium treatment of LX2 cells. Wound‐healing processes were recorded at 0, 6, 12, and 24 h to calculate LX2 cell migration. D,E) Immunofluorescence (IF) staining to assess fibrosis markers in LX2 cells or primary mouse hepatic stellate cells (pmHSCs) 48 h after conditioned medium treatment. F) Experimental design for examining in vivo impact of hepatocyte E4bp4 deficiency on the activation of pmHSCs upon acute CCl4 injection. G,H) pmHSCs were divided into three subgroups: quiescent, partially activated, and fully activated, based on the expression of α‐SMA and the protrusions of cells. pmHSC cell counts in different subgroups of *E4bp4^flox/flox^* (*n* = 4) versus *E4bp4‐LKO* (*n* = 4) mice; the percentage of fully activated and quiescent pmHSCs for *E4bp4^flox/flox^* and *E4bp4‐LKO*. I) *E4bp4^flox/flox^* (*n* = 3) versus *E4bp4‐LKO* (*n* = 3) mice were used to isolate pmHSCs following acute CCl4 injection. Cells were harvested for RT‐qPCR to measure the genes related to fibrosis and cell migration. The data were plotted as Mean ± SEM. ^*^ *p* < 0.05, ^**^ *p* < 0.01, ^***^ *p* < 0.001 by the Student's *t*‐test.

To uncover the mechanisms of how exactly hepatocyte *E4bp4* deficiency mitigates liver fibrosis, we reasoned that hepatocyte E4BP4 might impact the duration and degree of activation of HSCs, which are the central driver of liver fibrosis.^[^ ^7^ ^]^ To test this hypothesis, we designed a medium transfer experiment to examine the impact of hepatocyte E4BP4 on the activation of HSCs. Specifically, conditioned medium was collected from the human hepatocyte cell line Huh7 cells after transduction with Ad‐*E4bp4* versus Ad‐LacZ control and washing off the viruses. Then, a conditioned medium was used to culture the human stellate cell line LX2 cells for 48 h (**Figure** **3**A). With the conditioned medium from Ad‐*E4bp4*‐transduced hepatocytes, we detected elevated levels of α‐SMA, VIMENTIN, and phosphorylated ERK in LX2 cells by immunoblotting (Figure 3B), in line with accelerated cell proliferation and migration determined by wound‐healing assay (Figure 3C) as well as positive staining of α‐SMA and Vimentin by immunofluorescence (Figure 3D). To rule out that this was only specific to LX2, we performed a similar experiment in primary mouse hepatic stellate cells (pmHSCs) isolated from *C57BL/6* WT mice.^[^ ^46^ ^]^ Similar to our observations in LX2 cells, more staining of α‐SMA and Vimentin was detected in pmHSCs cultured in the conditional medium from Ad‐E4bp4‐transduced Huh7 cells (Figure 3E). Since both cell types were not in contact with each other during medium transfer, we concluded that hepatocyte E4BP4 is likely to promote the activation and fibrogenesis of HSC via secreting factor(s).

Based on the evidence from the medium transfer experiments, we further tested whether hepatocyte E4BP4 impacts HSC activation in vivo. To this end, we induced early‐onset activation of HSCs by intraperitoneally injecting WT versus *E4bp4‐LKO* mice with CCl4, before isolating pmHSCs. Immune‐staining for α‐SMA revealed three distinct groups of HSCs: quiescent HSCs (stained negative with α‐SMA), partially activated HSCs (staining positive for α‐SMA but lacking protrusions), and fully activated HSCs (positive α‐SMA signal with protrusions) (Figure S4, Supporting Information). In the four WT mice injected with CCl4, less than 2% HSCs were quiescent along with 20–40% HSCs partially activated and 60–80% HSCs fully activated. In contrast, *E4bp4‐LKO* mice showed 10% quiescent HSCs, 50% partially activated, and only 40% HSCs fully activated, which was significantly less than that of WT mice (Figure 3F–H). Moreover, we observed a significant reduction of the mRNA levels of markers of HSCs activation including *Acta2, Col1a1, Col1a2*, and *Mmp2* (Figure 3I). These results indicated that hepatocyte *E4bp4* deficiency creates an environment unfavorable for HSC activation in vivo. In summary, we provide both in vitro and in vivo evidence supporting the pro‐fibrogenic role of hepatocyte E4BP4 in the activation of HSCs in the liver.

### 2.4 E4BP4 Acts as a Positive Regulator of Hepatocyte OPN Expression

It has been observed that hepatocytes directly communicate with HSCs via a panel of secreting factors, including cytokine peptides, miRNA, and lipid molecules.^[^ ^12^ ^]^ To identify hepatocyte E4BP4‐specific factors that may activate HSCs, we performed RNA‐seq analysis of the liver samples of the 20‐week NASH diet‐fed *E4bp4‐LKO* cohort and revealed a number of secreted factors downregulated in the liver of NASH diet‐fed *E4bp4‐LKO* mice including *Bmp8b, Opn, Tgfb3, Fbn2, and Gdf15*.^[^ ^19^, ^47^, ^48^ ^]^

**Figure 4:**
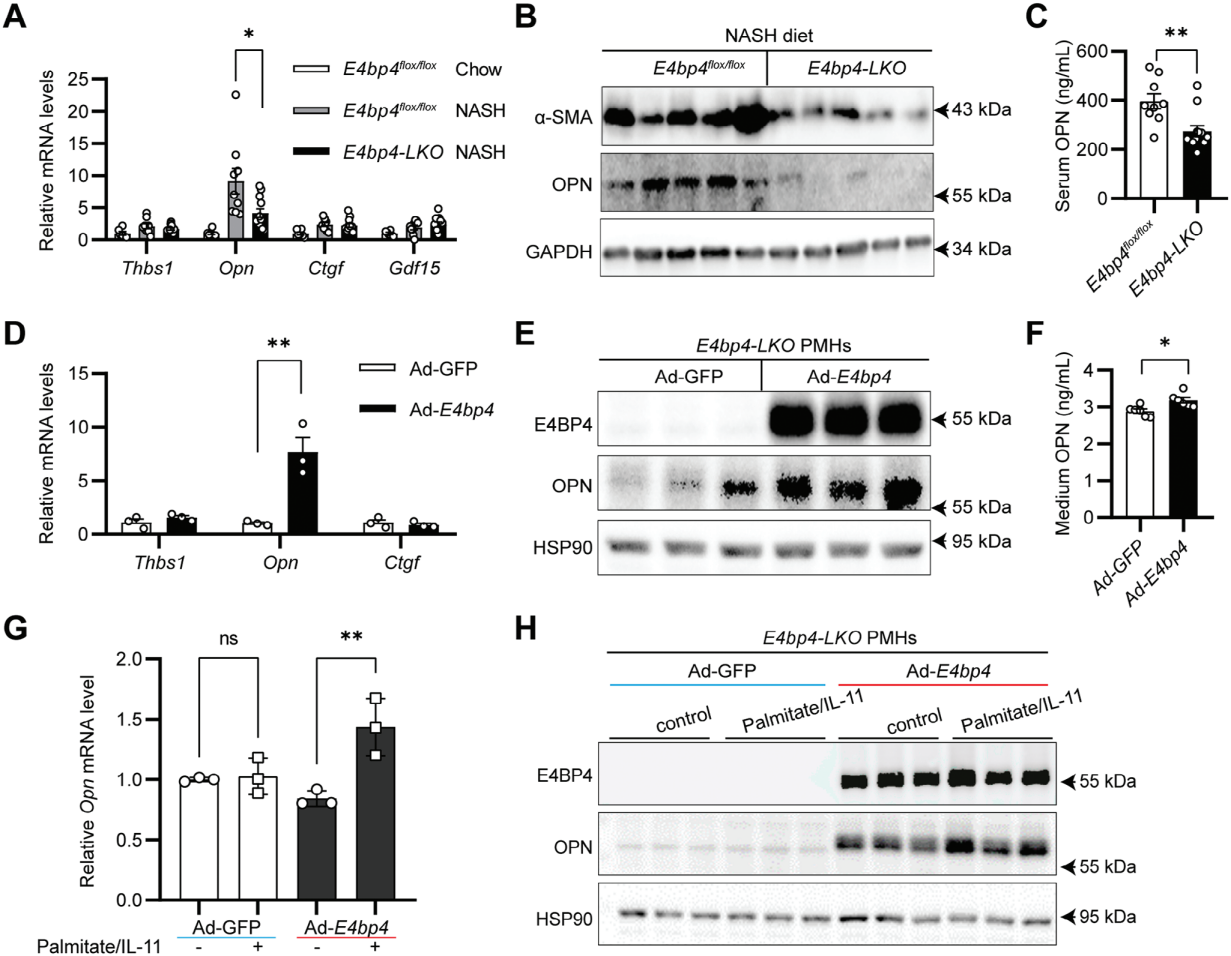
E4BP4 promotes hepatocyte OPN expression via a cell‐autonomous manner. Both 8‐week‐old *E4bp4^flox/flox^* male littermates (*n* = 9) and *E4bp4‐LKO* male mice (*n* = 12) were fed NASH diet 20 weeks. *E4bp4^flox/flox^* males on the chow diet were as control group (*n* = 6). A) Liver samples of the three groups of mice were used to assess the expression of pro‐fibrogenic genes by RT‐qPCR. B) Liver samples from NASH diet‐fed mice were used to assess the α‐SMA and OPN abundance by immunoblotting. C) Serum OPN levels of NASH diet‐fed mice were measured with ELISA. D,F) PMHs isolated from *E4bp4‐LKO* male mice were transduced with Ad‐GFP or Ad‐*E4bp4* for 24 h before RT‐qPCR to measure the expression levels of pro‐fibrogenic genes, 48 h before immunoblotting and ELISA to check the OPN protein abundance and its level in the medium. G,H) *E4bp4‐LKO* PMHs transduced with Ad‐LacZ or Ad‐*E4bp4* were cultured in serum‐free medium with 300 µm palmitic acid for 16 h plus IL‐11 (5 ng mL^−1^) for 6 h before *Opn* level detection by RT‐qPCR and immunoblotting. The data were plotted as Mean ± SEM. ^*^ *p* < 0.05, ^**^ *p* < 0.01 by the Student's *t*‐test for C, D, and F; by one‐way ANOVA for (A) and (G).

We next evaluated the expression dynamics of these factors during a time‐course experiment of NASH diet feeding. Our data showed that the mRNA levels of *Opn* and *Gdf15* were all induced by NASH diet at various time points. Interestingly we did not detect similar changes in *Bmp8b* and *Ctgf* even though these factors have been reported to be elevated in certain mouse models of NASH^[^ ^18^, ^38^, ^49^ ^]^ (Figure S5, Supporting Information). More importantly, hepatic *Opn* mRNA induction (about tenfold) by 20‐week MASH in *E4bp4^flox/flox^* was abrogated in *E4bp4‐LKO* mice (**Figure** **4**A). Meanwhile, the *Thbs1* and *Ctgf* expression was not impacted by hepatocyte *E4bp4* deficiency. Moreover, both liver protein and serum levels of OPN were markedly reduced in *E4bp4‐LKO* mice (Figure 4B,C), consistent with the reduced *Opn* mRNA in those mice.

To determine whether hepatocyte E4BP4 promotes *Opn* expression in a cell‐autonomous manner, we transduced primary mouse hepatocytes (PMHs) with Ad‐GFP versus Ad‐*E4bp4*. E4BP4 overexpression led to a significant increase of the *Opn* mRNA but not that of *Thbs1 and Ctgf* in PMHs (Figure 4D). Meanwhile, the OPN abundance in Ad‐*E4bp4*‐transduced hepatocytes as well as their culture medium was also elevated (Figure 4E,F).

Our data so far showed hepatocyte E4BP4 is required for induction of OPN in response to NASH diet feeding. To uncover the upstream signals that utilize E4BP4 to drive OPN overexpression in the context of NASH feeding, we transduced *E4bp4‐LKO* PMHs with Ad‐*E4bp4* versus Ad‐GFP and treated the cells with a combination of lipotoxicity (palmitate at 300 µm) and pro‐inflammatory cytokine (IL‐11 at 5 ng mL^−1^). Significant induction of the *Opn* mRNA and protein levels was only detected in Ad‐*E4bp4*‐transduced hepatocytes but not in Ad‐GFP‐transduced hepatocytes in response to palmitate plus IL‐11 treatment (Figure 4G,H). Taken together, we identified that hepatocyte E4BP4 is both sufficient and necessary for promoting OPN expression. Our data also support that hepatocyte E4BP4 is required for liver OPN induction following NASH diet, likely in response to nutritional and inflammatory stresses.

### 2.5 OPN Mediates HSCs Activation by Hepatocyte E4BP4

The profibrogenic actions of OPN are well‐documented.^[^ ^22^ ^]^ Nieto's group showed that OPN and HMGB1 drive the fibrogenic response to liver injury.^[^ ^26^ ^]^ Another study showed that OPN upregulates the integrin signaling in response to oxidative stress.^[^ ^24^ ^]^ OPN was also reported to play a crucial role in mediating the fibrogenic response of the NOTCH signaling during chronic high‐fat–high‐carbohydrate (HFHC) diet feeding.^[^ ^20^ ^]^ We thus hypothesized that hepatocyte E4BP4 promotes OPN production and secretion to activate HSCs, whereas downregulation of OPN impairs the E4BP4 ability to activate HSCs.

**Figure 5:**
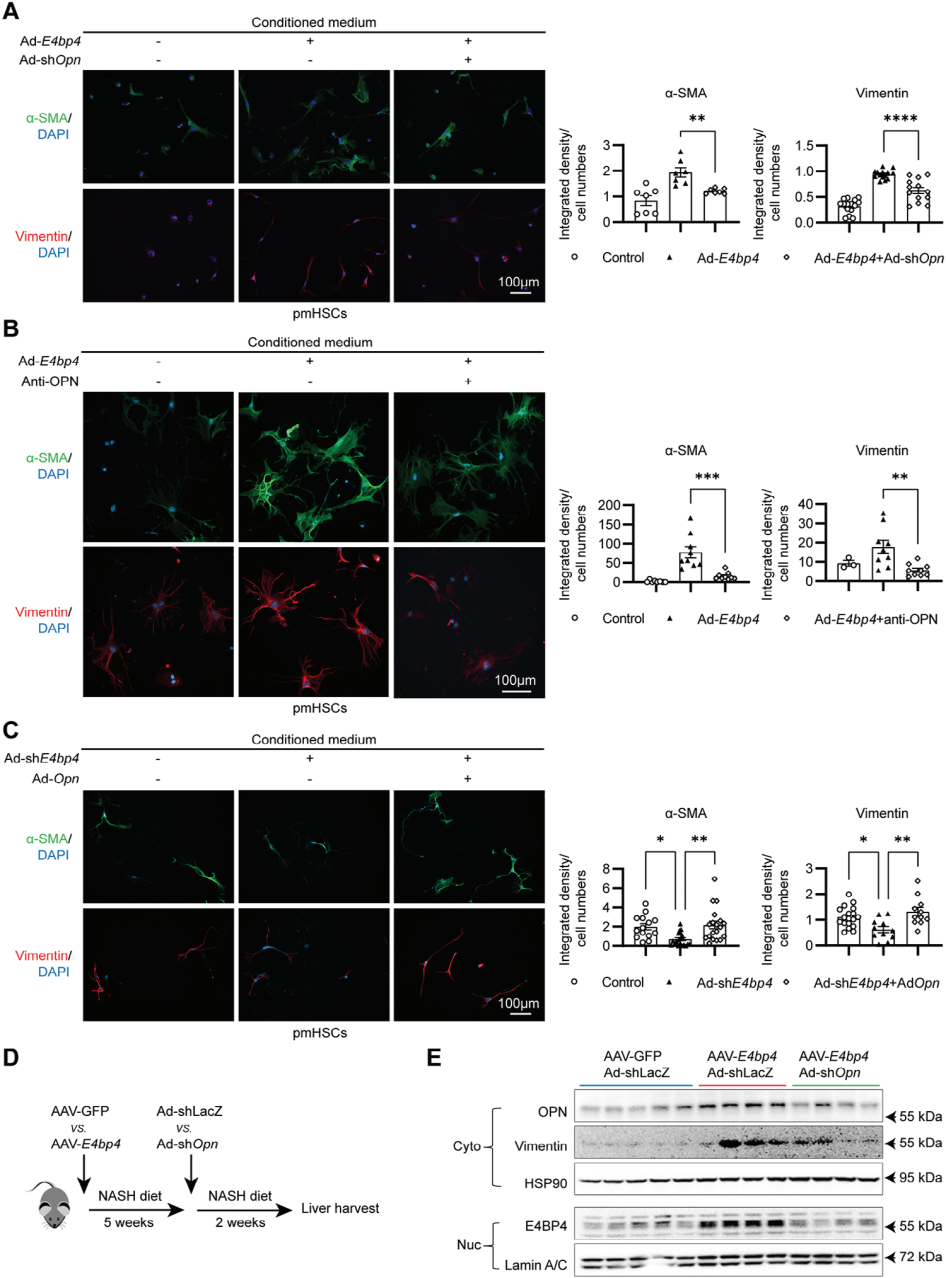
OPN mediates hepatocyte E4BP4‐induced HSC activation. A) Conditioned medium from Huh7 cells transduced with either Ad‐Control, Ad‐*E4bp4*, or Ad*E4bp4*/Ad‐sh*Opn* was used to culture pmHSCs prior to immunofluorescence staining with either anti‐α‐SMA or anti‐Vimentin. B) Conditioned medium collected from Huh7 cells transduced with either Ad‐Control *or* Ad*‐E4bp4* were incubated with anti‐OPN versus mouse IgG. Then neutralized medium was used to culture pmHSCs for 48 h prior to immunofluorescence staining with either anti‐α‐SMA or anti‐Vimentin. C) Conditioned medium from Huh7 cells transduced with either Ad‐Control, Ad‐sh*E4bp4* or Adsh*E4bp4*/Ad‐*Opn* was used to culture pmHSCs prior to immunofluorescence staining with either anti‐α‐SMA or anti‐Vimentin. D) Schematic of experimental design. E) 12‐week‐old WT male mice were injected with AAV8‐TBG‐GFP versus AAV8‐TBG‐*E4bp4* via the tail vein, then were subjected to NASH diet feeding for 5 weeks prior to injection of Ad‐sh*Opn* versus Ad‐shLacZ. NASH diet was continued for two additional weeks before sacrifice. The protein expression of E4BP4, OPN, and Vimentin were analyzed by immunoblotting. The data were plotted as Mean ± SEM.^*^ *p* < 0.05, ^**^ *p* < 0.01, ^***^ *p* < 0.001,^****^ *p* < 0.0001 by one‐way ANOVA.

To test this hypothesis, we used two independent approaches to block or deplete OPN production. We first generated Ad‐sh*Opn* to deplete *Opn* expression in the presence of E4BP4 overexpression. The efficiency of adenovirus‐mediated *Opn* knockdown and *E4bp4* overexpression was confirmed by immunoblotting in hepatocytes (Figure S6A, Supporting Information). Then, we collected the conditioned medium from these cells to culture pmHSCs for 48 h prior to immunofluorescence with anti‐α‐SMA and anti‐Vimentin. Consistent with previous results (Figure 3E), the conditioned medium from Ad‐*E4bp4*‐transduced hepatocytes markedly increased α‐SMA and Vimentin in pmHSCs, whereas the conditioned medium from *Opn*‐deleted hepatocytes reduced the two markers of HSC activation (**Figure** **5**A). Next, we employed an OPN‐specific antibody to neutralize OPN in the conditioned medium from Ad‐*E4bp4*‐transduced Huh7 cells prior to culturing stellate cells. The pull‐down specificity of anti‐OPN was confirmed by immunoprecipitation and immunoblotting in Huh7 cells after transduction with Ad‐*Opn* versus Ad‐LacZ (Figure S6B, Supporting Information). After incubation in the conditioned medium treated with anti‐OPN versus mouse IgG, pmHSCs showed significantly reduced staining of both α‐SMA and Vimentin (Figure 5B).

Lastly, we tested whether restoring OPN expression in *E4bp4*‐depleted hepatocytes enhances the activation of pmHSCs. *E4bp4* knockdown and *Opn* Overexpression were achieved by transducing hepatocytes with Ad‐sh*E4bp4* or Ad‐*Opn* and confirmed by immunoblotting (Figures S6C and S7A, Supporting Information). As shown in Figure 5C, *E4bp4*‐depleted conditioned medium lowered the intensity of α‐SMA and Vimentin in pmHSCs, whereas *Opn*‐enriched conditioned medium from Ad‐sh*E4bp4*‐transduced hepatocytes elevated both markers in pmHSCs (Figure 5C). Meanwhile, treating LX2 cells with the conditioned medium from hepatocytes with OPN overexpression lead to a robust increase in α‐SMA in LX2 cells by immunoblotting (Figure S7B, Supporting Information). Taken together, we accumulated evidence supporting that hepatocyte E4BP4 stimulates HSCs activation via inducing and releasing OPN into the liver microenvironment.

Next, we determined whether hepatocyte E4BP4‐mediated induction of OPN is conserved in mouse liver. To this end, we first generated hepatic overexpression of the E4BP4 model by injecting AAV8‐TBG‐*E4bp4* into WT mice and feeding mice NASH diet for 5 weeks before injecting mice with either Ad‐shLacZ or Ad‐sh*Opn* and continuing NASH diet for two additional weeks. AAV‐mediated E4BP4 overexpression indeed increased the protein levels of OPN and the fibrosis marker Vimentin. However, acute depletion of *Opn by* Ad‐sh*Opn* reduced Vimentin in the liver of Ad‐*E4bp4*‐injected mice. Unexpectedly, the nuclear E4BP4 protein was markedly reduced upon depletion of OPN, hinting at a potential feedback regulation of E4bp4 by OPN (Figure 5D,E). In summary, we provided both in vitro and in vivo evidence supporting hepatocyte E4BP4 in driving the profibrogenic factor OPN expression.

### 2.6 Hepatic E4BP4 Promotes OPN Expression via Upregulating YAP

How exactly E4BP4 promotes OPN induction remains unclear. Based on our previous work, E4BP4 could either function as a transcriptional repressor through direct binding to the promoters of its targets such as hepatic *Fgf21*,^[^ ^33^ ^]^ or act as a transcription coactivator via its interactions with other transcription factors, such as SREBP‐1c and CREBH.^[^ ^32^, ^35^ ^]^ We speculated that E4BP4 might activate the transcription of OPN indirectly by facilitating some known transcription factors of OPN. In the context of fibrosis, the *Opn* mRNA was known to be transactivated by YAP, RUNX2, SOX9, and C‐JUN in various cell types.^[^ ^20^, ^50^, ^51^, ^52^ ^]^

**Figure 6:**
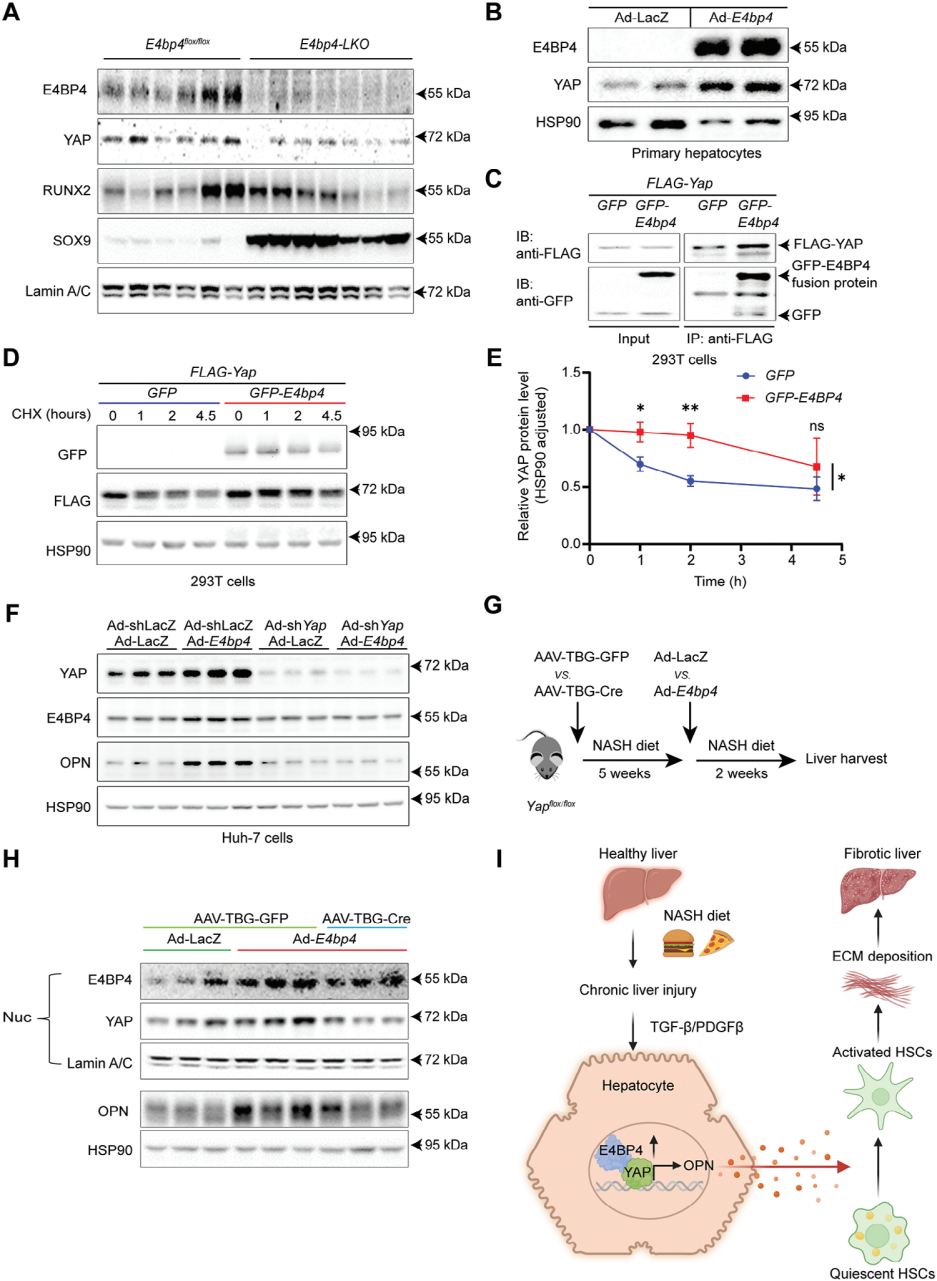
E4BP4 promotes OPN expression mainly via upregulating YAP. A) Both 8‐week‐old *E4bp4^flox/flox^* male littermates (*n* = 6) and *E4bp4‐LKO* male mice (*n* = 7) were fed with NASH diet for 20 weeks. Nuclear protein levels of the indicated protein in liver lysates were determined by immunoblotting. B) WT PMHs were transduced with AdGFP or Ad‐*E4bp4* for 48 h prior to the assessment of E4BP4 and YAP protein levels by immunoblotting. C) 293T cells were cotransfected with pCruz‐FLAG‐*Yap* along with pCruz‐GFP versus pCruz‐GFP‐*E4bp4* for 48 h. Cell lysates were used for immunoprecipitation with Flag‐beads. The presence of FLAG‐YAP, GFP, and GFP‐E4BP4 was detected by anti‐FLAG and anti‐GFP, respectively. D,E) 293T cells were cotransfected with pCruz‐FLAG‐*Yap* along with pCruz‐GFP versus pCruz‐GFP‐*E4bp4*. 36 h later, cells were treated with cycloheximide (100 µg/mL) for 0, 1, 2, and 4.5 h. The protein abundance of FLAG‐YAP and GFP‐E4bp4 was detected by immunoblotting. Three independent replicate experiments were performed. The data were plotted as mean ± SD. F) Huh‐7 cells were transduced with Ad‐shLacZ versus Ad‐sh*Yap* for 96 h and Ad‐*E4bp4* versus Ad‐GFP for 48 h prior to the assessment of the indicated protein levels by immunoblotting. G) Schematic illustration of experimental design. H) 12‐week‐old *Yap^flox/flox^* mice were injected with AAV‐TBG‐GFP versus AAV‐TBG‐Cre via the tail vein, then were subjected to NASH diet feeding for 5 weeks prior to Ad‐GFP versus Ad‐*E4bp4* injection. NASH diet was continued for 2 weeks before sacrifice. The protein levels of the indicated protein in liver lysates were detected by immunoblotting (*n* = 3, 3, 3). I) Schematic model depicting NASH diet‐induced liver fibrosis via E4BP4‐OPN axis. The data were plotted as Mean ± SEM. ^*^ *p* < 0.05, ^**^ *p* < 0.01, and ^***^ *p* < 0.001 by the Student's *t*‐test and two‐way ANOVA for 6E.

Interestingly, we found that only the nuclear abundance of YAP, but not RUNX2 and SOX9, was markedly downregulated in the liver of NASH diet‐fed *E4bp4‐LKO* mice (**Figure** **6**A). As a critical regulator of the Hippo pathway, YAP has been shown to be a key driver of MASH and liver fibrosis.^[^ ^53^, ^54^ ^]^ We demonstrated that Ad‐*E4bp4*‐mediated overexpression of E4BP4 in PMHs elevated YAP protein expression (Figure 6B) without affecting the *Yap* mRNA expression (Figure S8A, Supporting Information). Moreover, E4BP4 was found to interact with YAP when both proteins were overexpressed in 293T cells (Figure 6C), and significantly enhanced the protein stability of YAP in a cycloheximide chase experiment (Figure 6D,E). Intriguingly, overexpression of E4BP4 also enhanced the protein stability of YAP‐S127A mutant,^[^ ^55^, ^56^ ^]^ which is resistant to LATS‐mediated phosphorylation, suggesting that E4BP4 stabilizes YAP independently of the classical Hippo pathway (Figure S8B, Supporting Information).

To gain further insights into how E4BP4 promotes YAP stability, we evaluated the mRNA levels of several YAP‐specific E3 ligases, including *Herc3*,^[^ ^57^ ^]^ *Mib2*,^[^ ^58^ ^]^ *Btrc*,^[^ ^55^ ^]^ *Rnf31*,^[^ ^59^ ^]^ *Rnf146*,^[^ ^60^ ^]^ *and Skp2*.^[^ ^61^ ^]^ When compared with WT mice, the levels of *Mib2* and *RNF146* were significantly elevated in the liver of *E4bp4‐LKO* mice, indicating their possible involvement in degrading YAP protein in the absence of E4BP4 (Figure S8C, Supporting Information).

Next, we tested out how depletion of *Yap* impacts E4BP4‐induced OPN in hepatocytes. While Ad‐E4bp4 transduction led to a clear elevation of OPN in Huh7 hepatocytes, such induction was largely impaired in the *Yap*‐deleted hepatocytes (Figure 6F). Lastly, to further examine whether YAP is required for the induction of OPN by E4BP4 in the liver, we generated a mouse model with acute deletion of *Yap* in hepatocytes by tail vein injection *Yap^flox/flox^* mice with AAV‐TBG‐Cre versus AAV‐TBG‐GFP prior to a second round of injection with Ad‐*E4bp4* versus Ad‐LacZ control (Figure 6G). It is evident that Ad‐*E4bp4* plus AAV‐TBG‐GFP‐injected *Yap^flox/flox^* mice displayed an increase of E4BP4 and OPN in the liver, whereas Ad‐*E4bp4* plus AAV‐TBG‐Cre‐injected mice showed decreased YAP protein and no change in OPN despite increased E4BP4 (Figure 6H). Taken together, we uncovered that E4BP4 promotes hepatocyte OPN expression via stabilizing YAP protein in both cultured hepatocytes and the mouse liver (Figure 6I).

## 3 Discussion

Development from simple steatosis to steatohepatitis represents the critical juncture in MASLD progression because liver fibrosis and inflammation are the major determinants of disease prognosis and the prelude to cirrhosis and liver failure. Excessive activation of HSCs plays a key role in the progression of liver steatosis to fibrosis. It has been proposed that the pro‐fibrogenic microenvironment surrounding HSCs provides continuous fibrogenic stimuli to activate HSCs.^[^ ^12^ ^]^

As the dominant cell type within the liver, normal hepatocytes not only carry out major metabolic functions but also maintain a healthy intrahepatic microenvironment. However, very little is known about how stressed or injured hepatocytes impact the intrahepatic microenvironment and subsequently HSC activation. In this study, we show that upon chronic intake of a diet enriched in fructose, cholesterol, and saturated fat (NASH diet), WT mice develop the whole spectrum of MASH, including liver steatosis, inflammation, and fibrosis, whereas mice with hepatocyte *E4bp4* deficiency are protected from fibrosis and inflammation despite a similar degree of liver steatosis. Moreover, the expression of a key profibrogenic factor OPN is markedly downregulated in the liver of *E4bp4‐LKO* mice, while E4BP4 is both necessary and sufficient to promote OPN expression within hepatocytes. Manipulation of hepatic E4BP4 promotes HSCs activation in an OPN‐dependent manner. Thus, activation of the E4BP4‐OPN axis in hepatocytes during NASH diet feeding could alter the hepatic microenvironment to favor sustained activation of HSCs and promote fibrosis. Of note, hepatocytes can communicate with HSCs through a variety of secreted factors, including cytokine peptides, miRNAs, and lipid molecules.^[^ ^5^, ^62^ ^]^ Even though our in vitro and in vivo data provide evidence for the critical role of OPN in mediating the crosstalk between hepatocyte E4BP4 and HSCs, we will investigate the possible involvement of other peptides, miRNA, and/or lipid molecules in future studies.

We previously reported that hepatocyte *E4bp4* deficiency protects mice from hepatocyte injury or liver steatosis following 10‐week HFLMCD diet or 12‐week HFD, respectively.^[^ ^34^, ^35^ ^]^ In response to the HFLMCD diet, loss of hepatocyte *E4bp4* could offer protection by promoting sustained activation of the AMPK pathway.^[^ ^34^ ^]^ In the case of HFD, *E4bp4‐LKO* mice were most likely protected via the downregulation of hepatic *Fsp27β* and lipid droplet biogenesis.^[^ ^35^ ^]^ Interestingly, we did not observe similar responses in *E4bp4‐LKO* mice on the NASH diet. We also did not detect major differences in lipid metabolic genes of DNL, FAO, and lipid droplet binding proteins. Rather, we observed major differences in liver inflammatory and fibrosis signatures. These findings suggest an intriguing aspect of hepatocyte E4BP4 biology: Hepatocyte E4BP4 may respond to different diets with diet‐specific effects on liver metabolism, inflammation, and fibrosis. The specific responses might include post‐translational modifications of hepatic E4BP4. We previously reported that E4BP4 is a SUMOylated protein at five conserved lysine residues and its SUMOylation was significantly reduced in the liver of HFD‐fed WT mice.^[^ ^35^ ^]^ SUMOylation not only impacts the protein stability but also protein–protein interactions of its targets. It is possible that NASH diet feeding results in a specific SUMOylation pattern of E4BP4 and steers its action toward liver fibrosis. It is also fathomable that hepatocyte E4BP4 interacts with other transcription factors in a diet‐specific manner. We previously reported that E4BP4 interacts with nuclear SREBP‐1C in response to insulin or feeding to stimulate de novo lipogenesis.^[^ ^32^ ^]^ We also discovered that E4BP4 is also required for CREBH‐mediated activation of *Fsp27β* in the context of HFD feeding.^[^ ^35^ ^]^ Here we discovered that E4BP4 interacts with YAP to promote the OPN expression in response to NASH feeding. The exact input signals that dictate E4BP4‐YAP interaction during MASH remain to be uncovered. Given that liver‐targeted E4BP4 antagonists may be fraught with side effects including impaired liver NK cell and innate immunity, inhibition of the signal‐driven E4BP4‐YAP interaction might hold great potential in curbing over‐activation of hepatocyte E4BP4 in MASH patients without affecting the normal physiological actions of E4BP4.

Hepatic YAP is well known for being a pivotal regulator of HSC activation and tissue fibrosis.^[^ ^53^ ^]^ In animal studies, overactivation of YAP promotes liver fibrosis,^[^ ^38^ ^]^ whereas YAP inhibition does the opposite.^[^ ^63^, ^64^ ^]^ Within the liver, YAP expression was found in both hepatocytes and non‐parenchymal cells, including HSCs. Deletion of *Yap* in HSCs reverses liver fibrosis during NASH diet.^[^ ^65^, ^66^ ^]^ Hepatocyte‐specific *Yap* knockout mice are protected from insulin resistance‐induced liver fibrosis.^[^ ^67^ ^]^ Here we demonstrate that hepatocyte E4BP4 interacts with YAP and promotes its protein stability in hepatocytes and the liver. Reduced hepatic YAP expression in *E4bp4‐LKO* mice is consistent with reduced HSCs activation and liver fibrosis, suggesting that hepatocyte E4BP4 could modulate liver microenvironment via YAP to promote the activation of HSCs and overproduction of ECM. Disrupting the E4BP4‐YAP protein‐protein interaction may represent a more specific antifibrosis avenue to treat liver fibrosis. How exactly hepatic YAP mediates fibrogenic actions and what signaling pathways maintain the robust YAP activity during liver fibrosis will be a focus of future research.

There are several limitations to our study. For instance, our conclusions were derived primarily from mouse models fed a NASH‐inducing diet. Although such a diet induces obesity, liver steatosis, inflammation, and fibrosis, additional studies are necessary to determine whether this diet‐induced pathophysiology accurately mimics human MASH pathogenesis. Furthermore, there might be additional E4BP4‐regulated profibrogenic factors whose roles during the pathogenesis of MASH remain to be examined. For instance, our RNA‐seq data revealed several secreted factors whose levels were reduced in the liver of *E4bp4‐LKO* mice. Further work is necessary to examine the relative contribution of these factors to MASH and liver fibrosis. Lastly, we observed that profibrogenic signals such as TGF‐β and PDGF‐β enhance the E4BP4 protein but not its mRNA in isolated hepatocytes, indicating involvement of post‐translational modifications, and future work is required to delineate the signaling cascades that enhance the E4BP4 protein stability in response to fibrogenic stimuli. Nevertheless, we believe that our current data provide strong evidence to support the critical role and underlying mechanisms of hepatocyte E4BP4 in promoting MASH‐associated liver fibrosis.

## 4 Experimental Section

### Animal Experiments

All animal experiments received approval from the Institutional Animal Care and Use Committee at the University of Michigan Medical School. C57BL/6 mice were housed in 12‐h light/dark cycles with unrestricted access to food and water. *E4bp4^flox/flox^* Alb‐Cre (+) (*E4bp4‐LKO*) mice were generated as described previously.^[^ ^34^ ^]^ 8‐week‐old *E4bp4^flox/flox^* mice and their *E4bp4‐LKO* littermates were fed with NASH diet (40% kcal from fat, 20% kcal from fructose, 2% cholesterol; Research Diets, D09100310) for 20 weeks, with *E4bp4^flox/flox^* mice on chow diet serving as the control group. Body weights were monitored weekly. To generate adult‐onset hepatocyte *Yap* knockout mice, 12‐week‐old *Yap ^flox/flox^* mice were injected with AAV‐TBG‐GFP or AAV‐TBG‐Cre (1 × 10^10^ viral particles/mouse) via the tail vein and then placed on a NASH diet for 7 weeks.

### RNA‐Seq Analysis

Total RNA was extracted with RNeasy (QIAGEN, 74104) from livers of *E4bp4^flox/flox^* (*n* = 4) and *E4bp4‐LKO* mice (*n* = 4) fed with NASH diet for 20 weeks. The samples were sent to the BGI Genomics for RNA sequencing. Differentially expressed genes (DEGs) were identified using the DESeq2 algorithm, and expression differences between the NASH diet‐fed *E4bp4^flox/flox^* and *E4bp4‐LKO* mice were visualized with a heatmap generated by the R heatmap package. RNA‐seq data are accessible in the NCBI's Gene Expression Omnibus (GEO) database (GEO GSE223518).

### Cellular Immunofluorescence Staining

LX2 cells or pmHSCs were seeded on collagen‐coated slides in 12‐well plates. For immunofluorescence staining, 48 h post‐conditioned medium treatment, cells were fixed with cold methanol at 4 °C for 5 min, washed thrice with 1X PBS, and blocked with 10% goat serum in 1X PBS for 30 min. Primary antibodies (α‐SMA and Vimentin), 1:200 diluted in 1X PBS containing 1.5% goat serum, were applied overnight at 4 °C. Alexa Fluor‐conjugated secondary antibodies (1:300 diluted in 1X PBS with 1.5% goat serum) were then added for 1 h at room temperature in darkness. Slides were mounted using DAPI‐containing antifade reagent (Invitrogen, P36931). Fluorescence images were captured using a KEYENCE fluorescence microscope (BZ‐X810, Itasca, IL, USA), and immunostaining quantification was performed with *ImageJ* software.

### Statistical Analyses

Statistical analysis was performed using Prism version 9.0 (GraphPad Software, San Diego, CA, USA). Statistical significance was determined either by unpaired two‐tailed Student's *t*‐test for comparison between two groups or by one‐way or two‐way *ANOVA* with Tukey's or Dunnett's post hoc test for multiple‐group comparison. All results are given as Mean ± SEM. Results were considered statistically significant with *p* value <0.05.

Additional methods are provided in Supplementary Materials.

## Conflict of Interest

The authors declare no conflict of interest.

## Author Contributions

S.W., J.G., and M.Y. equally contributed to this work, carried out all the animal experiments, performed tissue analysis, analyzed the data, and generated figures. JS. G. also drafted the parts of the manuscript. SJ.W., JS.G., generated all the expression vectors and recombinant adenoviruses for in vitro and in vivo experiments with guidance from X.T., SJ.W., and JS.G. performed primary hepatocyte isolation, primary hepatic stellate cell isolation, immunofluorescence staining, and biochemical analysis. L.Y. and X.T. supervised the project and wrote the manuscript. Inputs and suggestions from all authors were incorporated.

## Supporting information

Supporting Information

## Data Availability

The data that support the findings of this study are openly available in Gene Expression Omnibus (GEO) at https://www.ncbi.nlm.nih.gov/geo/query/acc.cgi, reference number 223518.
