# Supplementary material for: OPN‐Mediated Crosstalk Between Hepatocyte E4BP4 and Hepatic Stellate Cells Promotes MASH‐Associated Liver Fibrosis
Source: Adv Sci (Weinh). 2024 Oct 29;11(47):2405678. doi: 10.1002/advs.202405678 (PMC11653607; doi:10.1002/advs.202405678)
Supplement: Supplementary file 1 — Supporting Information [file ADVS-11-2405678-s001.docx]

**Supplementary Figures**

**
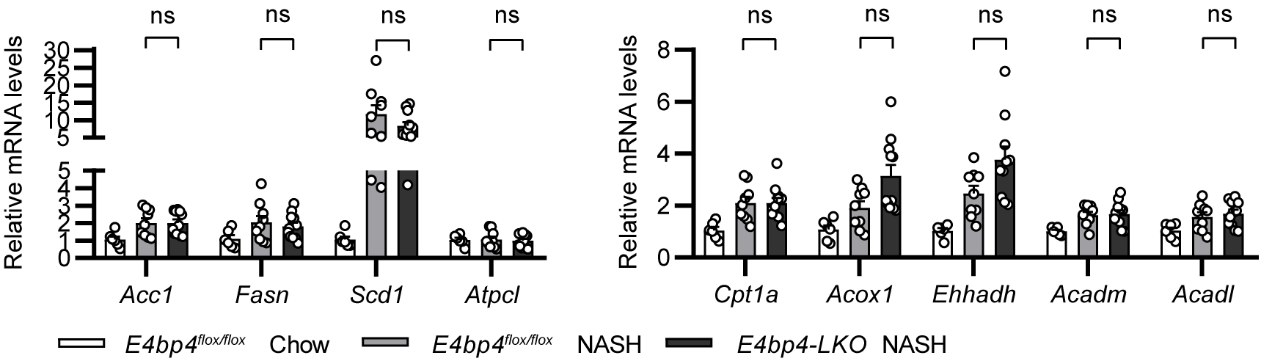
**

**Supplementary Figure S1 (Related to Figure 2). Hepatocyte *E4bp4* deficiency in NASH-diet fed mice has no effect on DNL- or FAO-related genes.** Both 8-week-old *E4bp4^flox/flox^*  male littermates (n = 9) and *E4bp4-LKO* male mice (n = 12) were fed with NASH diet for 20 weeks. *E4bp4^flox/flox^*  male mice on chow diet were as control group (n = 6). Liver samples of the three groups of mice were used to detect the expression levels of DNL- or FAO-related genes by RT-qPCR. The data were plotted as Mean ± SEM. Statistical significance was determined by one-way ANOVA.


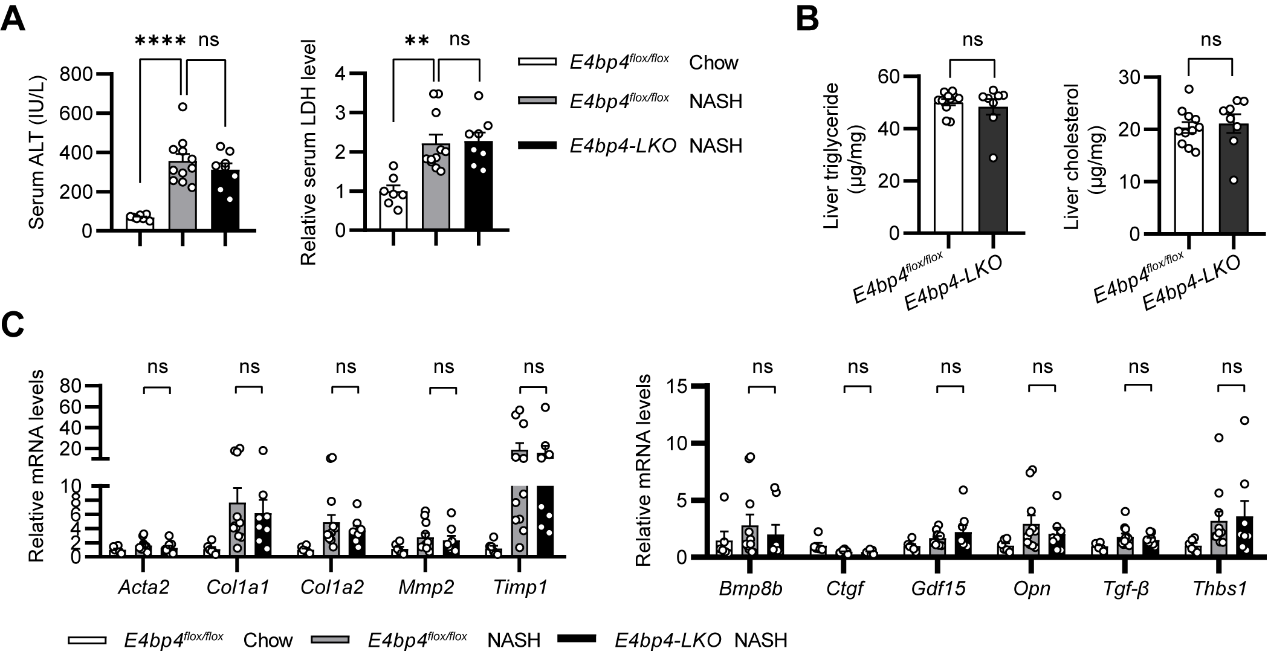


**Supplementary Figure S2 (Related to Figure 2). Hepatocyte *E4bp4* deficiency has no effect on NASH-diet fed female mice.** Both 8-week-old *E4bp4^flox/flox^*  female littermates (n=11) and *E4bp4-LKO* male mice (n = 8) were fed with NASH diet for 20 weeks. *E4bp4^flox/flox^*  male mice on chow diet were as control group (n=7). **(A)** Serum ALT and serum LDH levels; **(B)** liver triglyceride and cholesterol in NASH diet-fed mice; **(C)** fibrosis and fibrogenic genes detected by RT-qPCR.The data were plotted as Mean ± SEM. ***p* < 0.01, *****p* < 0.0001 by one-way ANOVA for A and C; by the Student’s t-test for B.


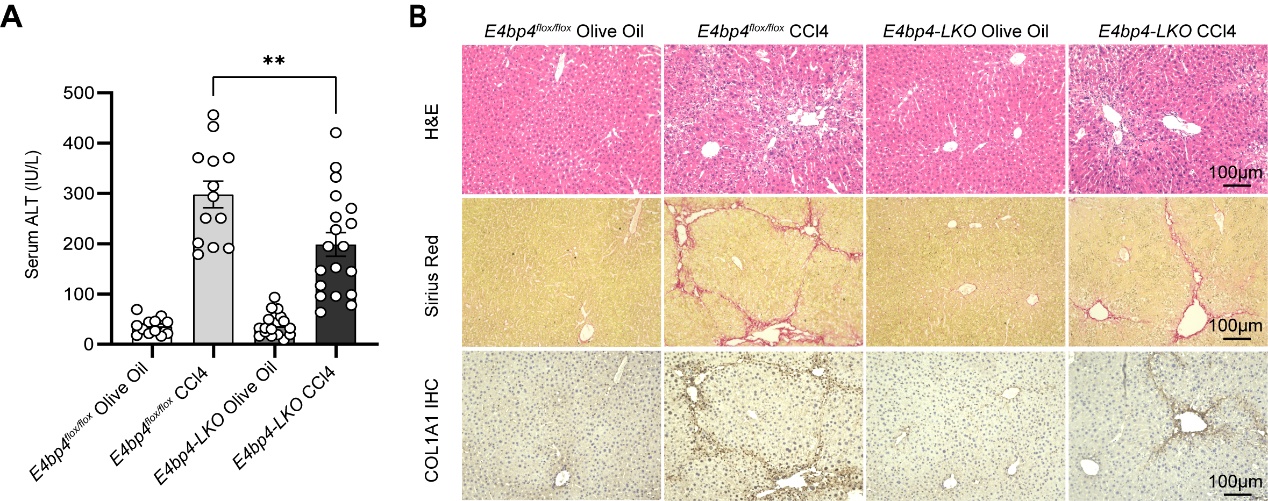


**Supplementary Figure S3 (Related to Figure 2). Hepatocyte *E4bp4* deficiency partially protects mice from CCl4-induced liver fibrosis.** Both 8-week-old *E4bp4^flox/flox^* mice (n=13) and their *E4bp4-LKO* littermates (n=20) were subjected to bi-weekly intraperitoneal injections of CCl4 (0.6 μL/g, diluted 1:9 in olive oil) for 8 weeks, *E4bp4^flox/flox^* (n=12) and *E4bp4-LKO* (n=12) mice injected with olive oil serving as control. (**A**) Serum ALT assay; (**B**) H&E staining, Sirius Red staining, and IHC with anti-COL1A1. The data were plotted as Mean ± SEM. ***p* < 0.01 by one-way ANOVA.


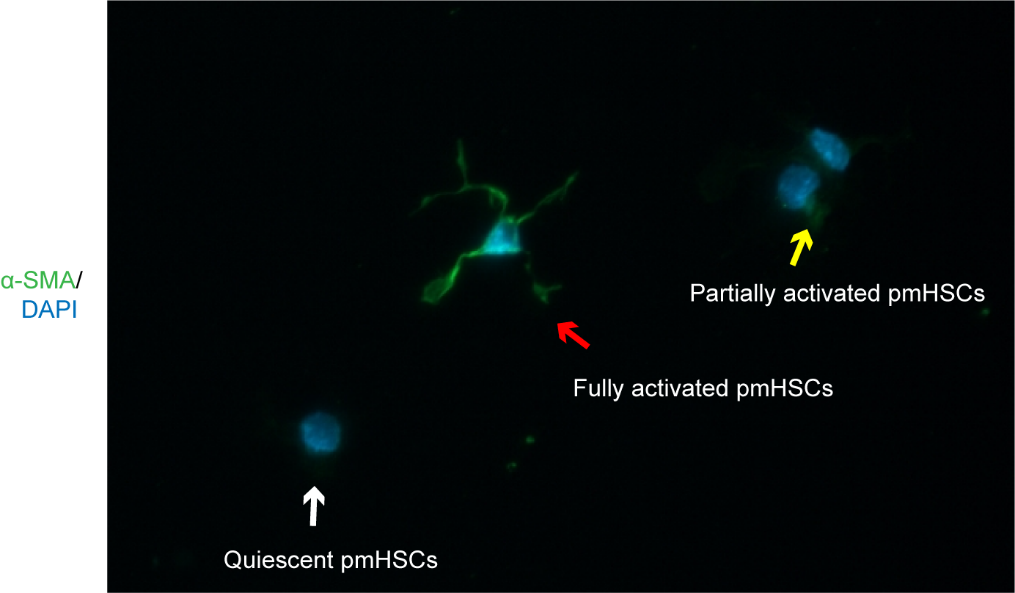


**Supplementary Figure S4 (Related to Figure 3). Three distinct hepatic stellate cell populations in response to CCl4-injecetd in mice.** No α-SMA immunofluorescent signal indicates quiescence (white arrow), light round signal suggests partial activation (yellow arrow), and bright branched signal denotes full activation (red arrow).


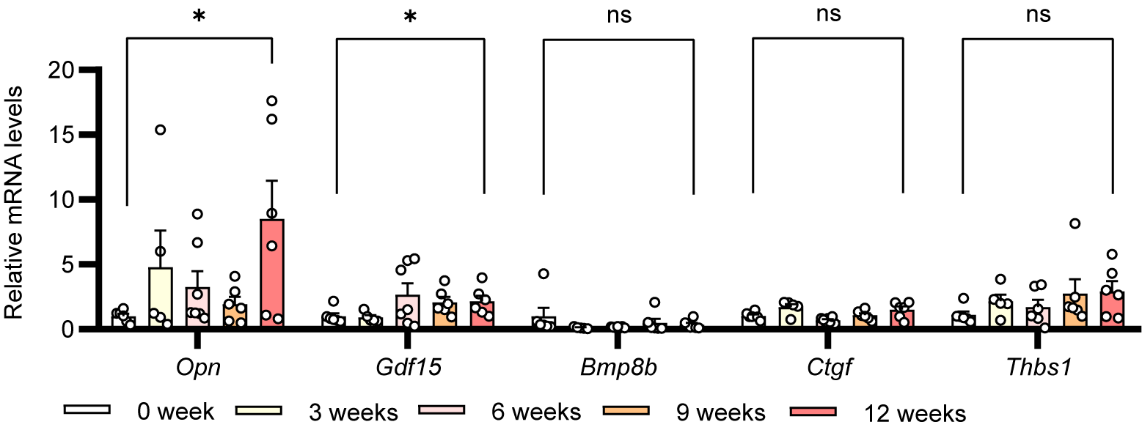


**Supplementary Figure S5 (Related to Figure 4).** **Effect of NASH diet feeding on the expression of profibrogenic genes in the liver.** 8-week C57BL/6 WT male mice were subjected to NASH diet for 0, 6, 9 and 12 weeks prior to dissection at the same age. Liver samples of those mice were used to detect the expression levels of profibrogenic genes by RT-qPCR. The data were plotted as Mean ± SEM. **p* < 0.05 by one-way ANOVA.


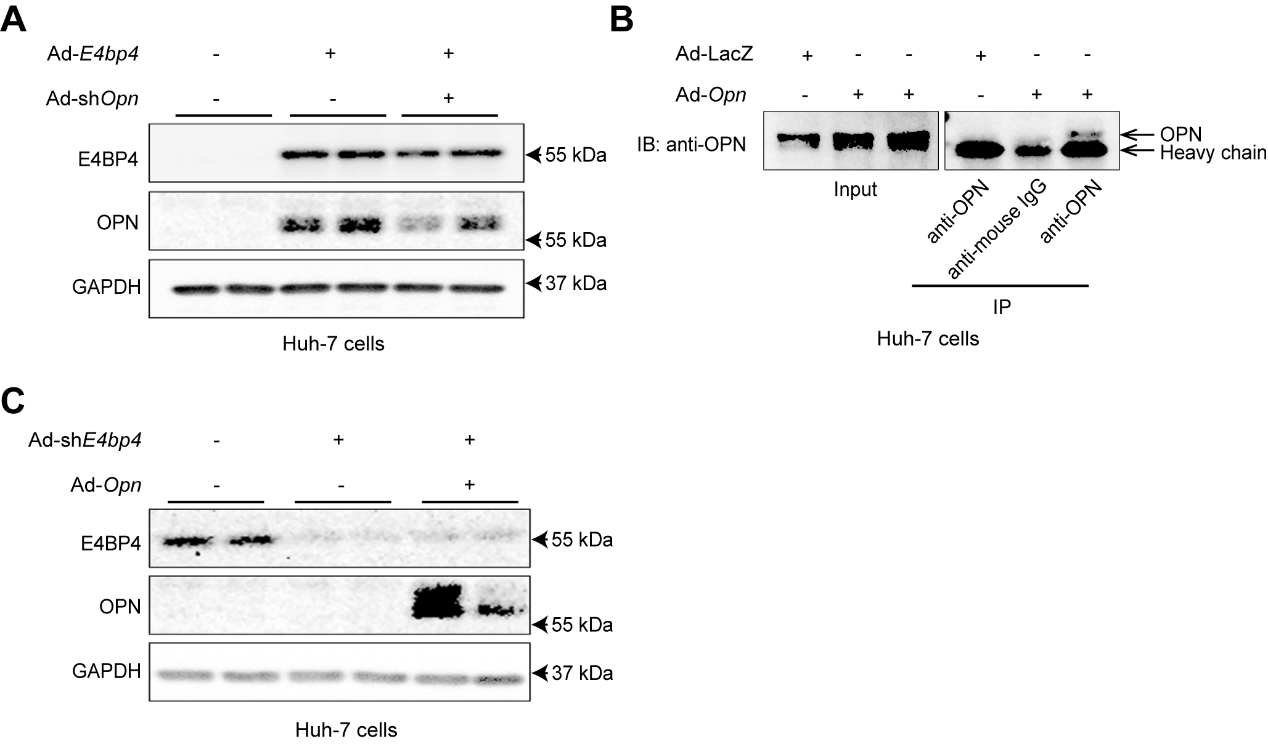


**Supplementary Figure S6 (Related to Figure 5). Validation of OPN depletion by adenoviral shRNA or anti-OPN antibody in hepatocytes.** (**A**) Huh-7 cells were transduced with Ad-sh*Opn* for 48 hours and Ad-*E4bp4* for 24 hours prior to immunoblotting, Ad-shLacZ and Ad-LacZ serving as control. (**B**) Huh-7 cells were transduced with Ad-LacZ or Ad*Opn* for 24 hours. Cell lysates were incubated with either anti-mouse IgG control or anti-OPN overnight and then used for immunoprecipitation with Protein A Sepharose beads. The presence of OPN was detected using anti-OPN by immunoblotting. (**C**) Huh-7 cells were transduced with Ad-sh*E4bp4* for 48 hours and Ad-*Opn* for 24 hours prior to immunoblotting. Ad-shLacZ and Ad-LacZ were included as control.


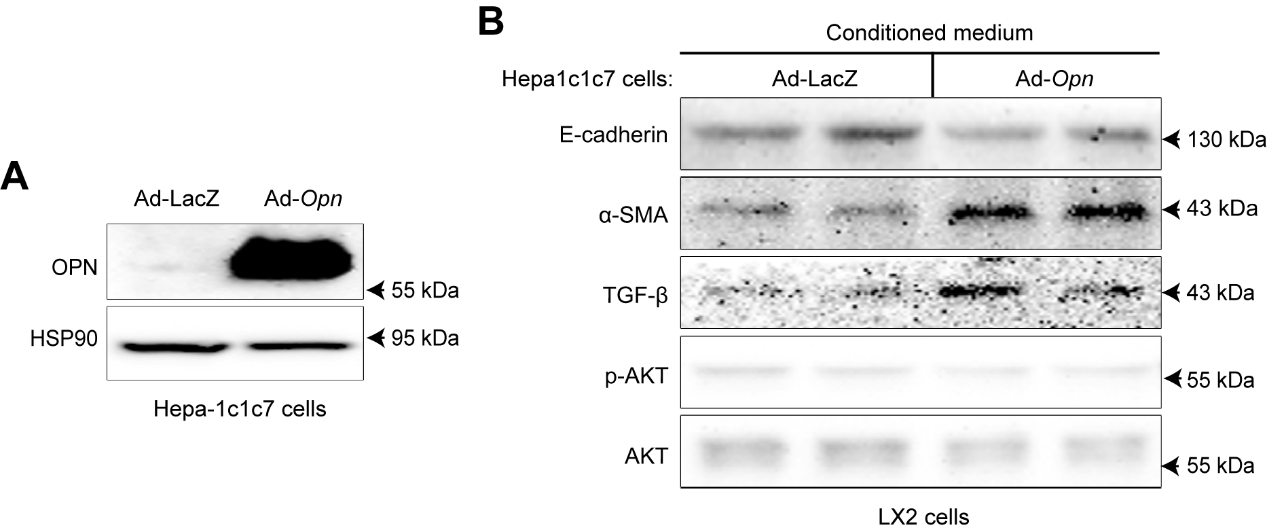


**Supplementary Figure S7 (Related to Figure 5). Hepatocyte-derived OPN promotes LX2 cell activation in vitro.** 8 hours after transduction with Ad-LacZ or Ad-*Opn,* Hepa-1c1c7 cells were washed with 1X PBS and then switched to serum-free medium. 24 hours after transduction, conditioned medium was collected to treat LX2 cells for 48h. (**A**) Hepa-1c1c7 cell were harvest for immunoblotting to verify virus-mediated OPN overexpression. (**B**) LX2 cells were collected for immunoblotting to assess fibrosis markers 48 hours post conditioned medium treatment.


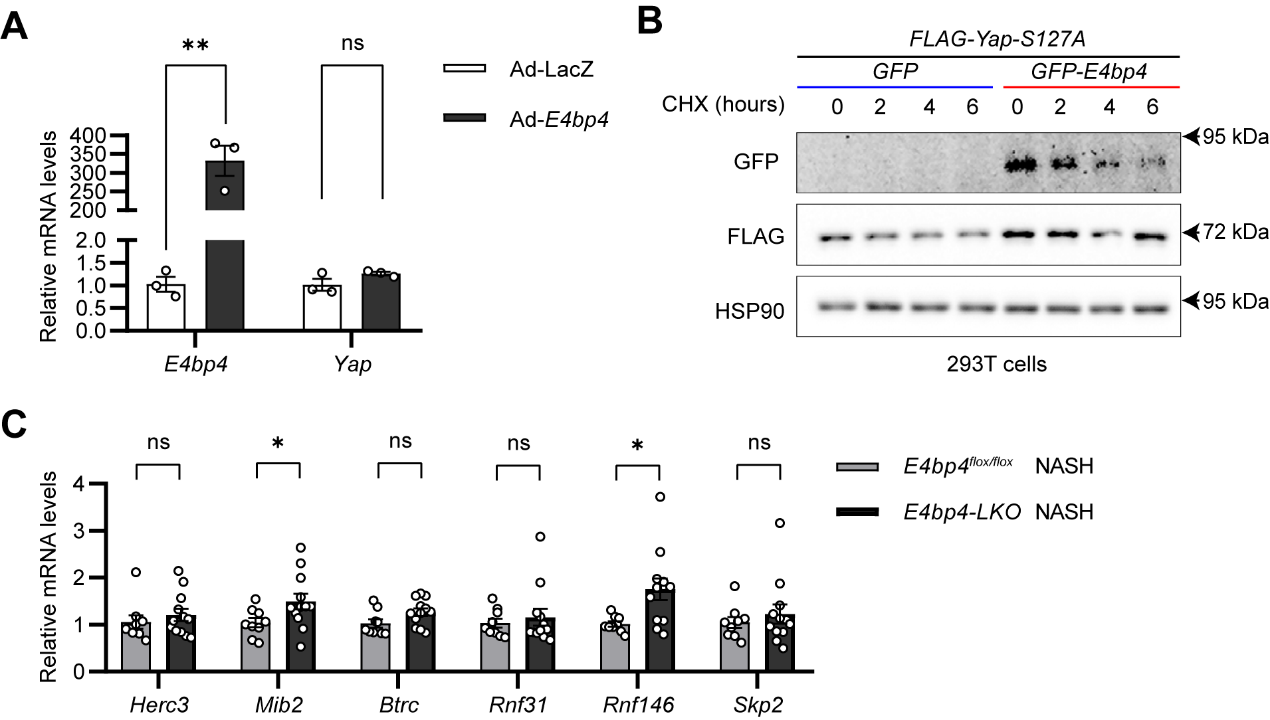


**Supplementary Figure S8 (Related to Figure 6).** ***E4bp4* regulates the YAP stability at the post-translational level.** (**A**) WT PMHs were transduced with Ad-GFP or Ad-*E4bp4* for 24 hours prior to the assessment of *E4bp4* and *Yap* mRNA levels by RT-qPCR. (**B**) 293T cells were co-transfected with pCruz-FLAG-Yap-S127A mutant along with pCruz-GFP *vs.* pCruz-GFP-*E4bp4* for 36 hours. Then, cells were treated with cycloheximide (100ug/ml) for 0, 2, 4, and 6 hours. The protein abundance of FLAG-YAP and GFP-E4bp4 was detected by immunoblotting. (**C**) Livers from 8-week-old *E4bp4^flox/flox^*  male mice (n=9) and their *E4bp4-LKO* male littermates (n = 12) fed with NASH diet for 20 weeks, were used to determine genes expression of YAP-specific E3 ligases by RT-qPCR. The data were plotted as Mean ± SEM. **p* < 0.05 , ***p* < 0.01 by the Student’s *t*-test.
